# A bibliometric analysis of polymyxin-induced nephrotoxicity research: Trends and insights (2005–2024)

**DOI:** 10.1097/MD.0000000000050976

**Published:** 2026-10-16

**Authors:** Wenjuan Duan, Shizhuo Liu, Zexuan Tian, Linan Wang, Yutian Zhang, Yingchao Ma, Xiuling Yang

**Affiliations:** a Department of Pharmacy, The Second Hospital of Hebei Medical University, Shijiazhuang, Hebei, China; b Graduate School of Hebei Medical University, Shijiazhuang, Hebei, China.

**Keywords:** bibliometric analysis, CiteSpace, nephrotoxicity, polymyxins, VOSviewer

## Abstract

**Background:**

Polymyxins remain important therapeutic options for the treatment of multidrug-resistant Gram-negative bacterial infections; however, their clinical application is limited by a narrow therapeutic window and a high risk of nephrotoxicity. Although research on polymyxin-associated nephrotoxicity has expanded rapidly in recent years, the overall evolution of research themes, knowledge structure, and emerging frontiers in this field remains unclear. This study aimed to systematically evaluate the development trends and research hotspots of polymyxin-induced nephrotoxicity using bibliometric analysis.

**Methods:**

Relevant publications on polymyxin-induced nephrotoxicity published between January 1, 2005, and November 13, 2024, were retrieved from the Web of Science Core Collection database. CiteSpace and VOSviewer (Baxter Healthcare Corporation) were used to analyze publication trends, authorship, institutional and country collaboration networks, keyword co-occurrence, clustering, and burst detection.

**Results:**

A total of 569 publications were included in this study. The annual publication output demonstrated an overall increasing trend, indicating increasing attention to polymyxin-associated nephrotoxicity research. Li Jian was the most productive author, Monash University was the most productive institution in terms of publication output, and the United States contributed the highest number of publications among all countries. Keyword analysis revealed that research themes have evolved from early characterization of nephrotoxicity and clinical safety assessment toward the identification of acute kidney injury risk factors, elucidation of oxidative stress-related mechanisms, pharmacokinetic/pharmacodynamic optimization, and development of individualized dosing strategies and nephroprotective approaches. Burst analysis further indicated increasing attention toward biomarkers, precision dosing, and novel therapeutic strategies.

**Conclusion:**

Bibliometric analysis revealed that research on polymyxin-induced nephrotoxicity has progressively evolved from toxicity characterization to mechanistic exploration and precision management. Current challenges include an incomplete understanding of interactions among nephrotoxic pathways, limited dynamic prediction models, and insufficient clinical translation of emerging protective strategies. Future research should focus on integrating multi-omics approaches, artificial intelligence-based risk prediction, therapeutic drug monitoring, and safer polymyxin formulations to optimize the balance between antibacterial efficacy and nephrotoxicity prevention.

## 1. Introduction

The global increase in infections caused by multidrug-resistant (MDR) Gram-negative bacteria has become a major challenge in clinical antimicrobial therapy. Meanwhile, the limited availability of effective treatment options has further intensified the difficulty of managing these infections. Among currently available antimicrobial agents, polymyxins have regained considerable clinical attention as important therapeutic options for severe infections caused by carbapenem-resistant Enterobacterales, carbapenem-resistant *Acinetobacter baumannii*, and carbapenem-resistant *Pseudomonas aeruginosa*.^[1–6]^ Owing to their unique mechanism of disrupting bacterial outer membranes, polymyxins exhibit potent activity against a broad range of MDR Gram-negative pathogens and remain an essential component of antimicrobial strategies for these difficult-to-treat infections.

However, the clinical application of polymyxins is limited by their narrow therapeutic window and substantial risk of nephrotoxicity. Previous studies have identified nephrotoxicity as one of the most frequent and clinically significant adverse effects associated with polymyxin therapy. A systematic review and meta-analysis involving 48 studies and 6199 adult patients reported that the incidence of nephrotoxicity associated with colistimethate sodium and polymyxin B was approximately 48% and 38%, respectively.^[7,8]^ The difference in toxicity profiles between these 2 agents may be attributed to their distinct pharmacokinetic characteristics. Colistimethate sodium is a prodrug that requires conversion to the active form, colistin, within the body, whereas polymyxin B is administered directly as an active compound.^[9]^ Furthermore, excessive drug exposure has been associated with an increased risk of nephrotoxicity, and the exposure range required for optimal antibacterial efficacy may overlap with that associated with renal injury.^[10]^ Therefore, achieving an appropriate balance between antimicrobial effectiveness and nephrotoxicity prevention remains a major challenge in the clinical use of polymyxins.

In recent years, research on polymyxin-associated nephrotoxicity has expanded rapidly, covering multiple aspects including epidemiological characteristics, clinical risk factors, molecular mechanisms of renal injury, pharmacokinetic/pharmacodynamic (PK/PD)-guided dosing strategies, biomarkers, and nephroprotective interventions. Although numerous studies have contributed to advances in understanding polymyxin-induced nephrotoxicity, a comprehensive evaluation of the evolution of research topics, knowledge structure, collaboration patterns, and emerging trends within this field remains lacking.

Bibliometric analysis is a quantitative approach that enables systematic evaluation of scientific development patterns by identifying influential contributors, mapping knowledge networks, and revealing research hotspots and emerging frontiers. Therefore, this study employed bibliometric methods to analyze publications related to polymyxin-induced nephrotoxicity from 2005 to 2024. The specific objectives were as follows: to characterize publication trends and development patterns in this field; to identify collaboration networks among leading authors, institutions, and countries; to explore major research themes and their evolution through keyword co-occurrence and clustering analyses; and to identify emerging research directions through keyword burst analysis. By constructing a comprehensive knowledge map, this study aims to provide an objective overview of the intellectual landscape and evolutionary trajectory of polymyxin-induced nephrotoxicity research, identify potential knowledge gaps, and provide insights for future investigations and optimization of clinical management strategies.

## 2. Method

### 2.1. Data sources and search strategy

This study used the Web of Science Core Collection (WoSCC) database as the primary source for literature retrieval. The selection of this database was based on several considerations. WoSCC provides standardized bibliographic records, comprehensive citation data, and consistent author and institutional information, which are essential for rigorous bibliometric analysis. In addition, the data structure of WoSCC is highly compatible with bibliometric analysis tools, such as CiteSpace and VOSviewer, facilitating co-occurrence analysis, collaboration network mapping, and knowledge graph construction. Although the use of a single database may result in the omission of some relevant studies and potential selection bias, WoSCC remains a widely recognized and commonly used data source in bibliometric research, providing a reliable and systematic data foundation for the present study.

An advanced topic search was performed in WoSCC using the following strategy: TS = ([“polymyxin B” OR “colistin” OR “polymyxin E” OR “Aerosporin” OR “Colistimethate Sodium” OR “colistin methanesulfonate” OR “CMS”] AND [“nephrotoxicity” OR “AKI” OR “renal injury” OR “kidney injury” OR “ARF” OR “renal toxicity” OR “kidney toxicity”]).The search period ranged from January 1, 2005, to November 13, 2024. The starting year of 2005 was selected because this period coincided with renewed clinical interest in polymyxins due to the increasing prevalence of MDR Gram-negative bacterial infections, which subsequently promoted extensive research on polymyxin efficacy, PK/PD, and associated nephrotoxicity. The endpoint of November 2024 was selected to include the most recent available publications at the time of data collection, allowing a comprehensive evaluation of research development over approximately 2 decades. The initial search identified a total of 1022 records.

### 2.2. Inclusion and exclusion criteria

To ensure the rigor and reproducibility of the literature screening process, inclusion and exclusion criteria were predefined before screening.

Studies were included if they met the following criteria: focused on polymyxin-associated nephrotoxicity; were published in English; were original research articles; were indexed in the WoSCC database; and contained complete bibliographic information, including author and institutional details.

Studies were excluded if they met any of the following criteria: unrelated to polymyxin-induced nephrotoxicity; non-original publications, including books, news reports, editorials, and letters; duplicate records; or studies lacking essential bibliographic information.

### 2.3. Literature screening process

The literature screening process was performed according to the predefined inclusion and exclusion criteria. The selection procedure consisted of 3 sequential stages.

First, all retrieved records were screened based on language criteria. Among the 1022 records initially identified from the WoSCC database, 15 non-English publications were excluded, leaving 1007 records for further assessment.

Second, the remaining records were screened according to publication type. A total of 66 non-original publications, including reviews, books, news reports, editorials, and letters, were excluded. After this step, 941 records remained for subsequent eligibility assessment.

Third, the remaining records were evaluated according to the predefined eligibility criteria. The titles, abstracts, and available bibliographic information of the records were assessed to determine their relevance to polymyxin-induced nephrotoxicity and their suitability for bibliometric analysis. During this stage, 372 records were excluded for the following reasons: duplicate records (n = 54), studies unrelated to polymyxin-induced nephrotoxicity (n = 187), and records lacking essential bibliographic information required for bibliometric analysis (n = 131). All screening decisions were made according to the predefined inclusion and exclusion criteria to maintain consistency throughout the selection process.

Finally, 569 articles meeting all eligibility criteria were included in the bibliometric analysis. The detailed literature selection process is illustrated in the PRISMA flow diagram (Fig. 1).

**Figure 1.**
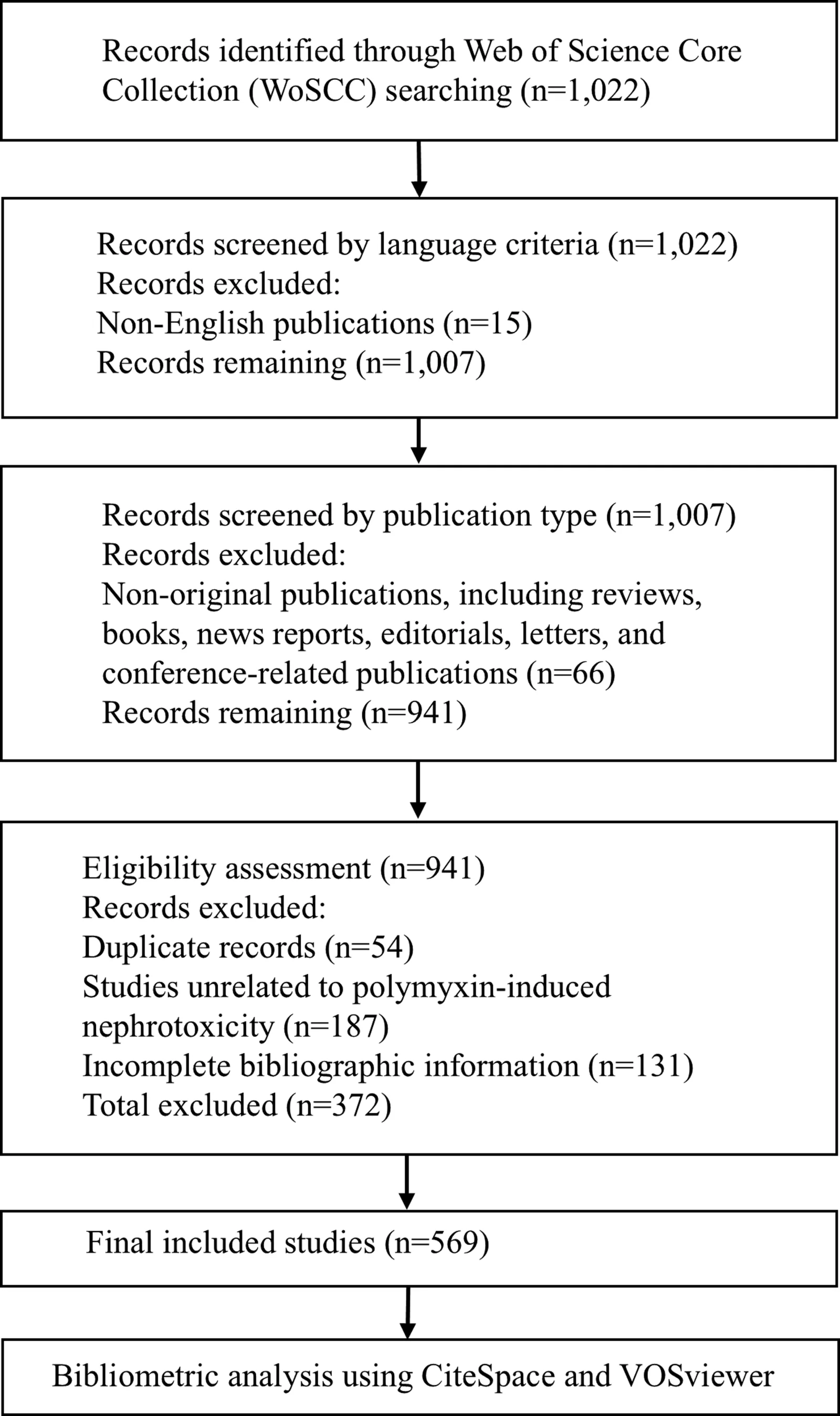
Data screening flowchart. n = number of records, WoSCC = Web of Science Core Collection.

### 2.4. Data processing and bibliometric analysis

The retrieved records were exported from the WoSCC database in RefWorks format and subjected to data preprocessing, including data format conversion, duplicate removal, and exclusion of records lacking essential bibliographic information. The standardized dataset was subsequently used for bibliometric analysis.

This study employed CiteSpace and VOSviewer to construct scientific knowledge maps and visualize research patterns. CiteSpace was used for analyses of collaboration networks, keyword clustering, keyword burst detection, and reference co-citation analysis. The analysis period was set from January 2005 to November 2024 with a 1-year time slice. The selected node types included authors, institutions, countries/regions, keywords, and references.

The g-index algorithm was applied for node selection with k = 25. The g-index was selected because it considers both publication productivity and citation impact, allowing influential nodes to be identified while maintaining network interpretability. The value of k = 25 was determined based on preliminary testing to achieve a balance between retaining important information and avoiding excessive network complexity. The thresholds were set as Top N = 50 and Top N% = 10%. Other parameters were kept consistent across analyses.

In this study, centrality was used to evaluate the structural importance of nodes within collaboration and co-occurrence networks. A higher centrality value indicates that a node occupies a more influential position within the network and may serve as a bridge connecting different research groups, topics, or knowledge domains. The quality and reliability of clustering results were evaluated using the modularity *Q* value and silhouette score.

VOSviewer was used to perform bibliographic coupling, co-authorship analysis, and keyword co-occurrence analysis. Authors, institutions, and countries/regions were selected as units of analysis for collaboration network construction, while keywords were analyzed to identify major research topics and emerging hotspots. The generated networks were visualized to explore the knowledge structure, collaboration patterns, and thematic evolution of polymyxin-induced nephrotoxicity research.

### 2.5. Ethical statement

As this study was based solely on publicly available literature data and did not involve human participants, identifiable patient information, or animal experiments, ethical approval was not required.

## 3. Results

### 3.1. Annual publication volume

A total of 1022 records were initially identified from the WoSCC database. After sequential screening based on language, publication type, and eligibility criteria, 569 articles were finally included in the bibliometric analysis. The detailed literature selection process is presented in Figure 1.

Between 2005 and 2024, a total of 569 publications on polymyxin-induced nephrotoxicity were included in the analysis. As shown in Figure 2, the annual publication output in this field demonstrated an overall increasing trend.

**Figure 2.**
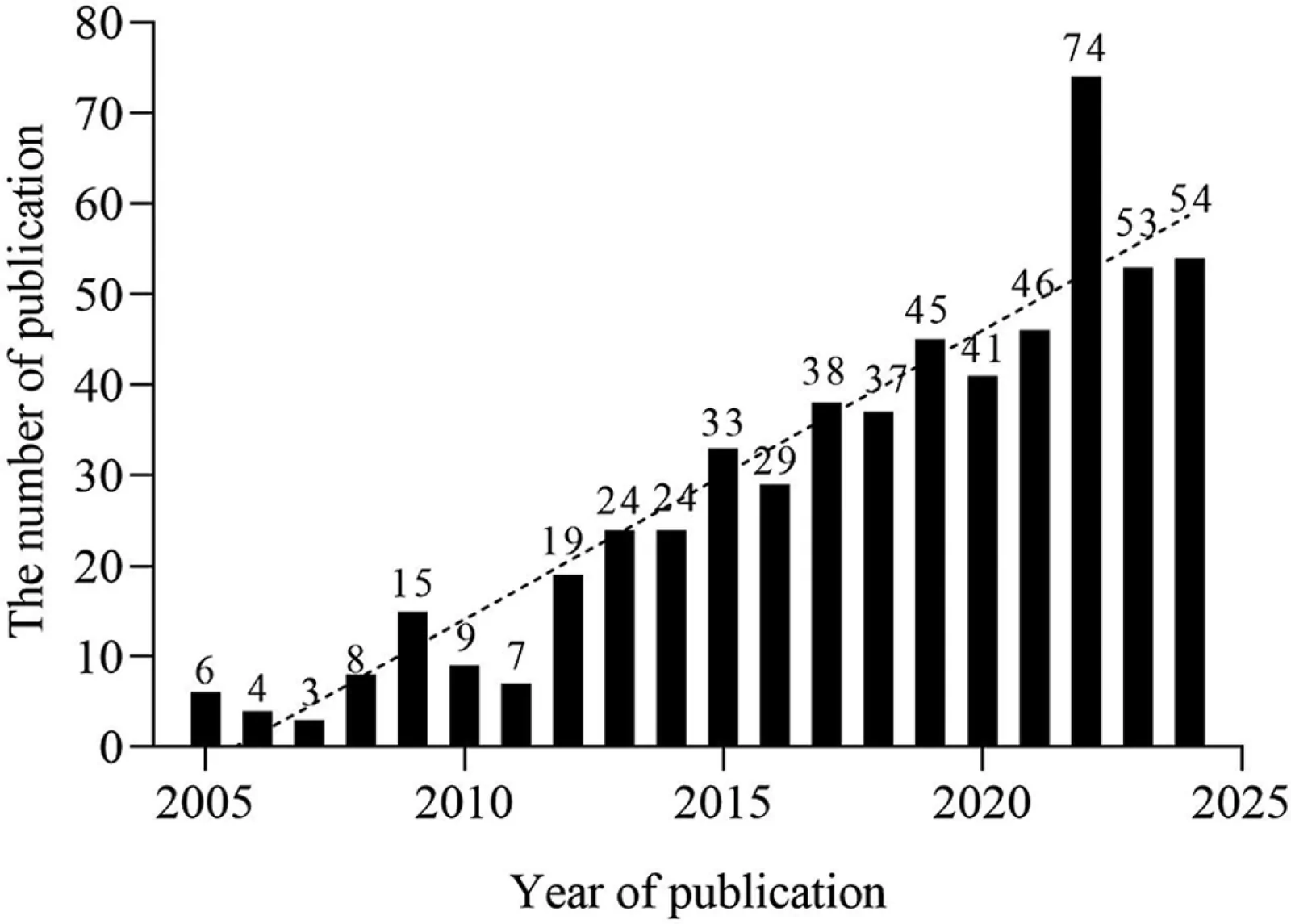
Annual publication volume from 2005 to 2024.

### 3.2. Coauthor

A total of 3174 authors were identified as contributing to research on polymyxin-induced nephrotoxicity. Based on publication output, the 3 most prolific authors were Li Jian (34 publications), Roger L. Nation (26 publications), and Tony Velkov (21 publications). According to Price Law, the threshold for identifying core authors was calculated based on the publication output of the most productive author (N_max = 34). The resulting threshold was 4.37 publications; therefore, authors with at least 5 publications were considered core authors. A total of 28 core authors were identified, who collectively contributed 276 publications, accounting for 48.5% of all publications in this field. Table 1 presents the top 10 authors ranked by publication output. Among these highly productive authors, Roger L. Nation had the highest average number of citations per publication, with 80 citations per publication. The author collaboration networks are presented in Figure 3A and 3B, illustrating collaboration patterns among authors identified using CiteSpace and VOSviewer, respectively.

**Table 1:** Top 10 authors in polymyxin-induced nephrotoxicity research.

Rank	Author	Publications	Citations	Average citations/publication
1	Li Jian	34	2121	62
2	Roger L Nation	26	2068	80
3	Tony Velkov	21	967	46
4	Kade D Roberts	13	522	40
5	Mohammad A K Azad	12	501	42
6	Alexandre P Zavascki	12	780	65
7	Vincent H Tam	11	495	45
8	Philip E Thompson	11	453	41
9	Martti Vaara	11	540	49
10	Wasan Katip	10	224	22
11	Peninnah Oberdorfer	10	222	22

**Figure 3.**
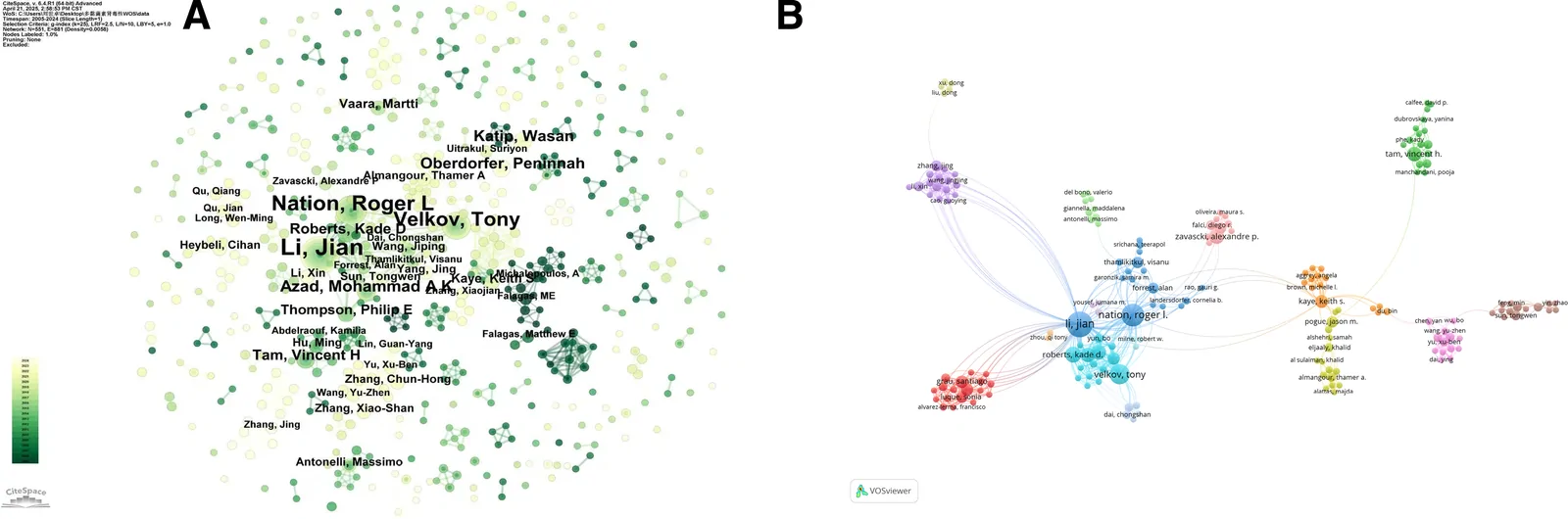
Author collaboration analysis. (A) Co-authorship network constructed using CiteSpace; (B) co-authorship network constructed using VOSviewer.

### 3.3. Co-institution

Institutional collaboration in the field of polymyxin-induced nephrotoxicity was analyzed. The institutional collaboration networks generated by CiteSpace and VOSviewer are presented in Figure 4A and 4B, respectively. Monash University ranked first in publication output, with 40 publications. Its centrality in the institutional collaboration network was 0.22. Zhejiang University, the Chinese Academy of Medical Sciences, Peking Union Medical College, and Central South University followed, each contributing 12 publications. Differences in network centrality were observed among institutions, with Monash University demonstrating the highest centrality within the collaboration network.

**Figure 4.**
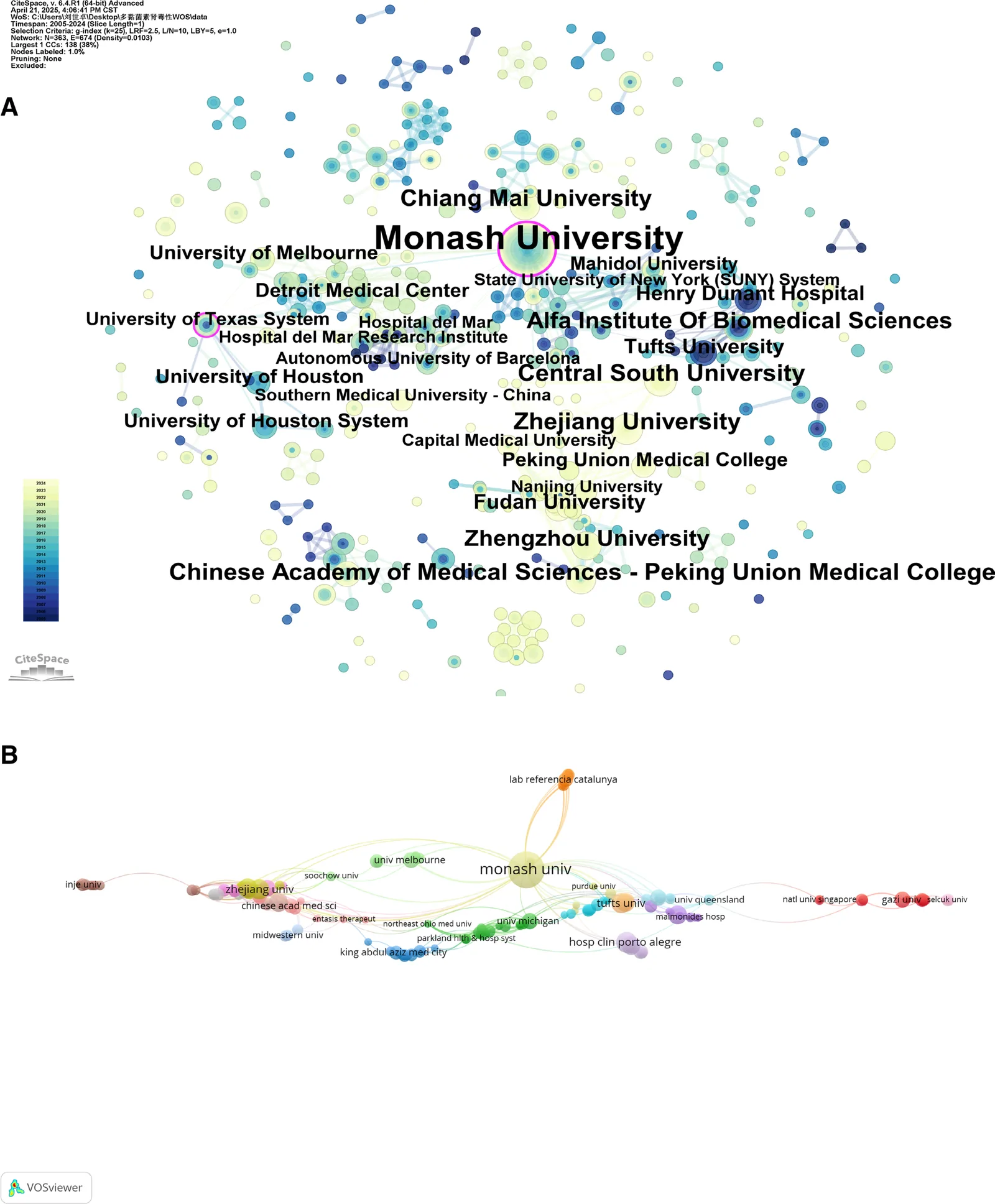
Institutional collaboration networks. (A) Institutional co-authorship network generated by CiteSpace; (B) institutional co-authorship network generated by VOSviewer.

### 3.4. Co-country

A bibliometric analysis was performed on the top 10 countries ranked by publication output, as shown in Table 2. The United States, China, and Türkiye were the 3 most productive countries, contributing 123, 122, and 52 publications, respectively. Among the top 10 countries, the United States (0.54), Australia (0.11), and Thailand (0.10) showed relatively high centrality values within the international collaboration network. Greece ranked eighth in terms of publication output, with 25 publications, but demonstrated the highest average number of citations per publication (112 citations per publication). The global distribution of publications and international collaboration networks are presented in Figure 5A and 5B.

**Table 2:** Top 10 countries contributing to polymyxin-induced nephrotoxicity research.

Rank	Country	Publications	Citations	Average Citations/publication	Centrality
1	United States	123	7174	58	0.54
2	China	122	1736	14	0.05
3	Türkiye	52	1269	24	0.05
4	Australia	49	2727	56	0.11
5	Brazil	30	1450	48	0.05
6	South Korea	29	656	23	0.01
7	Thailand	27	699	26	0.1
8	Greece	25	2803	112	0.05
9	Italy	22	1490	68	0.08
10	Saudi Arabia	17	268	16	0.03

**Figure 5.**
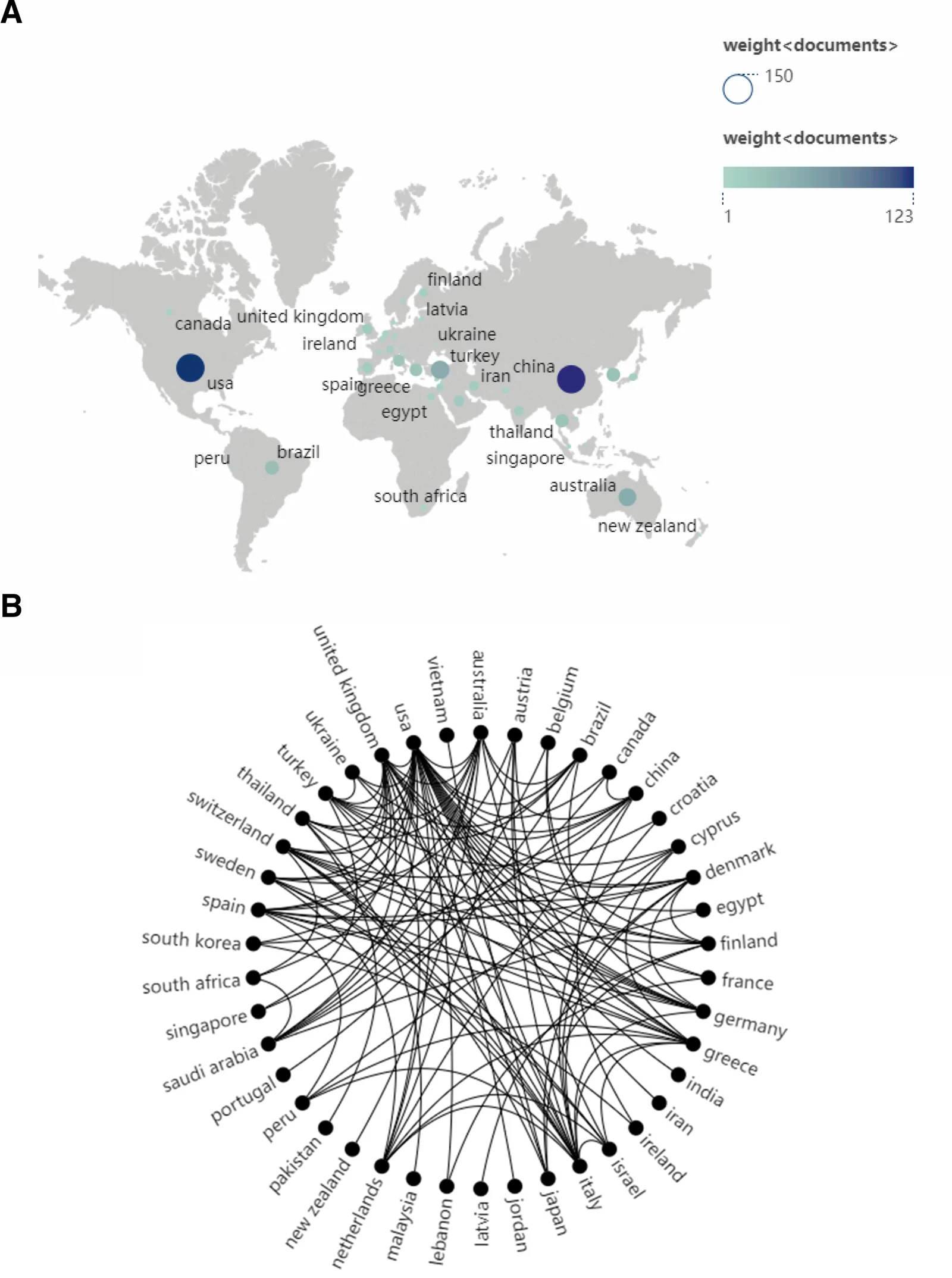
Country analysis of polymyxin-induced nephrotoxicity research. (A) Distribution of publications among different countries; (B) international collaboration network among countries.

### 3.5. Keywords

#### 3.5.1. Keywords co-occurrence

Keyword co-occurrence analysis was performed based on the retrieved records from the WoSCC database. The resulting keyword network consisted of 201 nodes and 5928 links. As shown in Table 3, the most frequently occurring keywords were “colistin” (356 occurrences), “nephrotoxicity” (263 occurrences), and “polymyxin B” (165 occurrences). Centrality analysis indicated that “colistin,” “acute kidney injury,” and “acute renal failure” were among the highly connected keywords. Notably, “colistin” and “acute renal failure” showed the highest centrality values, both with a centrality of 0.14. The keyword co-occurrence networks generated by CiteSpace and VOSviewer are presented in Figure 6A and 6B, respectively.

**Table 3:** Top 10 keywords of polymyxin-induced nephrotoxicity frequency and centrality.

Rank	Keywords	Frequency	Rank	Keywords	Centrality
1	colistin	356	1	colistin	0.14
2	nephrotoxicity	263	2	acute renal failure	0.14
3	polymyxin b	165	3	gram-negative bacteria	0.12
4	acute kidney failure	163	4	colistimethate sodium	0.11
5	critically-ill patients	155	5	*Acinetobacter baumannii*	0.1
6	Intravenous colistin	141	6	antibiotics	0.1
7	infections	106	7	toxicity	0.09
8	*Pseudomonas aeruginosa*	97	8	colistimethate	0.08
9	methanesulfonate	97	9	oxidative stress	0.08
10	risk factors	95	10	combination therapy	0.07

**Figure 6.**
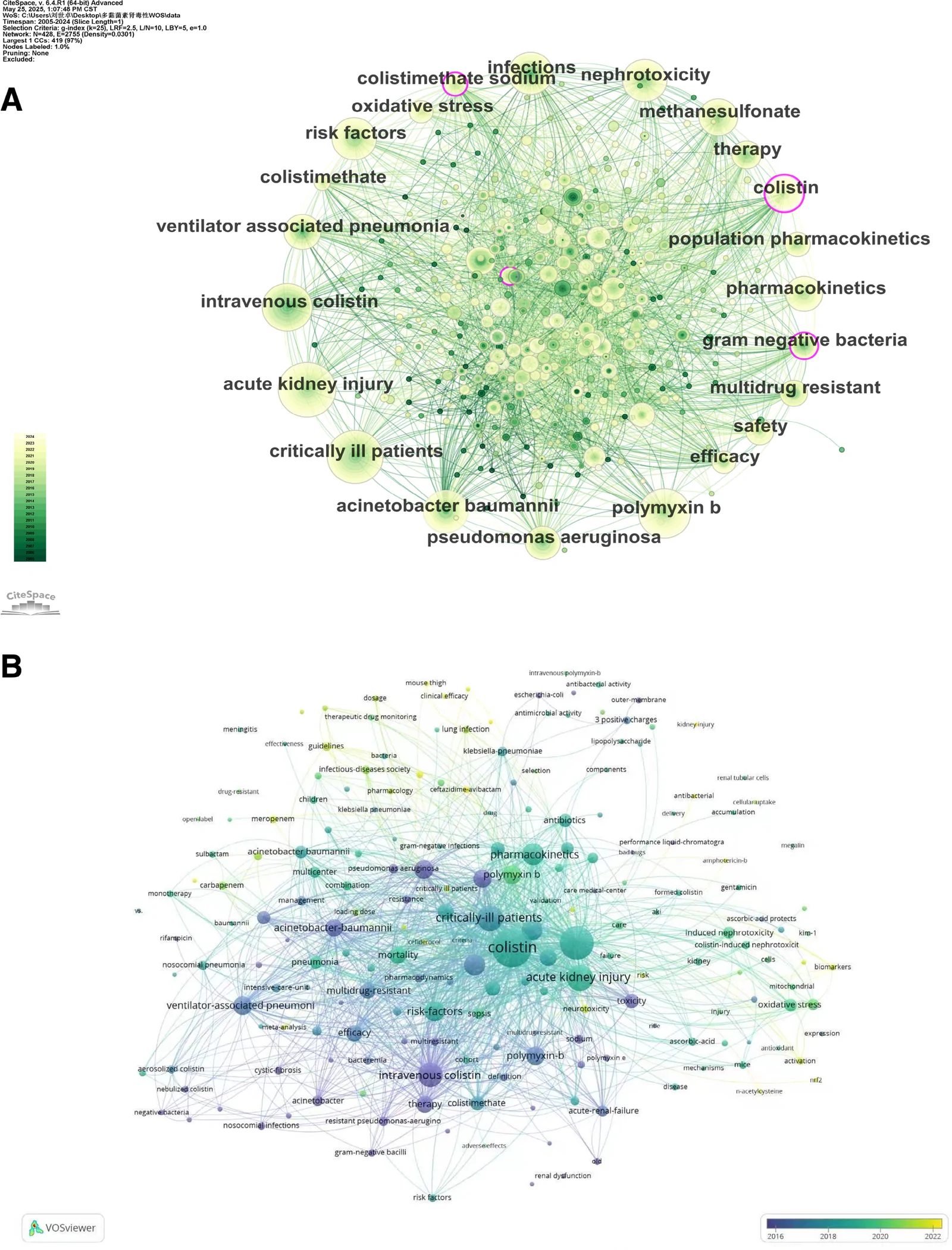
Keyword co-occurrence analysis. (A) Keyword co-occurrence network constructed using CiteSpace; (B) keyword co-occurrence network constructed using VOSviewer.

#### 3.5.2. Keywords clusters

The keyword clustering analysis identified 9 clusters (#0–#8). The cluster network and landscape visualization generated by CiteSpace are presented in Figure 7A and 7B, respectively. The clusters were labeled as follows: #0 acute kidney injury, #1 oxidative stress, #2 *Escherichia coli*, #3 *Acinetobacter baumannii* infection, #4 ventilator-associated pneumonia, #5 renal outcomes, #6 multidrug resistance, #7 colistimethate sodium pharmacokinetics, and #8 extensively drug-resistant *A baumannii*. Detailed information on each cluster, including cluster size and major labeling terms, is presented in Table 4.

**Table 4:** Keyword clustering results of polymyxin-induced nephrotoxicity research.

Cluster ID	Size	Cluster	Clustering label words (LLR)
#0	76	acute kidney injury	bacteremia; *Pseudomonas aeruginosa*; polymyxin b; antibiotics
#1	74	oxidative stress	apoptosis; curcumin; ascorbic acid; inflammation
#2	52	escherichia coli	antibiotics; antimicrobial activity; enterobacteriaceae; carbapenem resistance
#3	52	*Acinetobacter baumannii* infection	acute kidney injury; ventilator-associated pneumonia; intravenous; aerosolized colistin
#4	47	ventilator-associated pneumonia	loading dose colistin; renal tubular cells; resistant *Acinetobacter baumannii*; combination therapy
#5	39	outcome	renal dysfunction; epidemiology; acute renal failure; sodium deoxycholate sulfate
#6	36	multidrug resistance	intraventricular polymyxin b; *Pseudomonas aeruginosa*; intensive care unit; gram-negative bacteria
#7	27	methanesulfonate	multi-resistant performance liquid chromatography; colistin plasma concentrations; creatinine clearance
#8	16	extensively drug-resistant *Acinetobacter baumannii*	CRE; treatment; extensively drug-resistant; effectiveness

CRE = carbapenem-resistant Enterobacterales, LLR = log-likelihood ratio.

**Figure 7.**
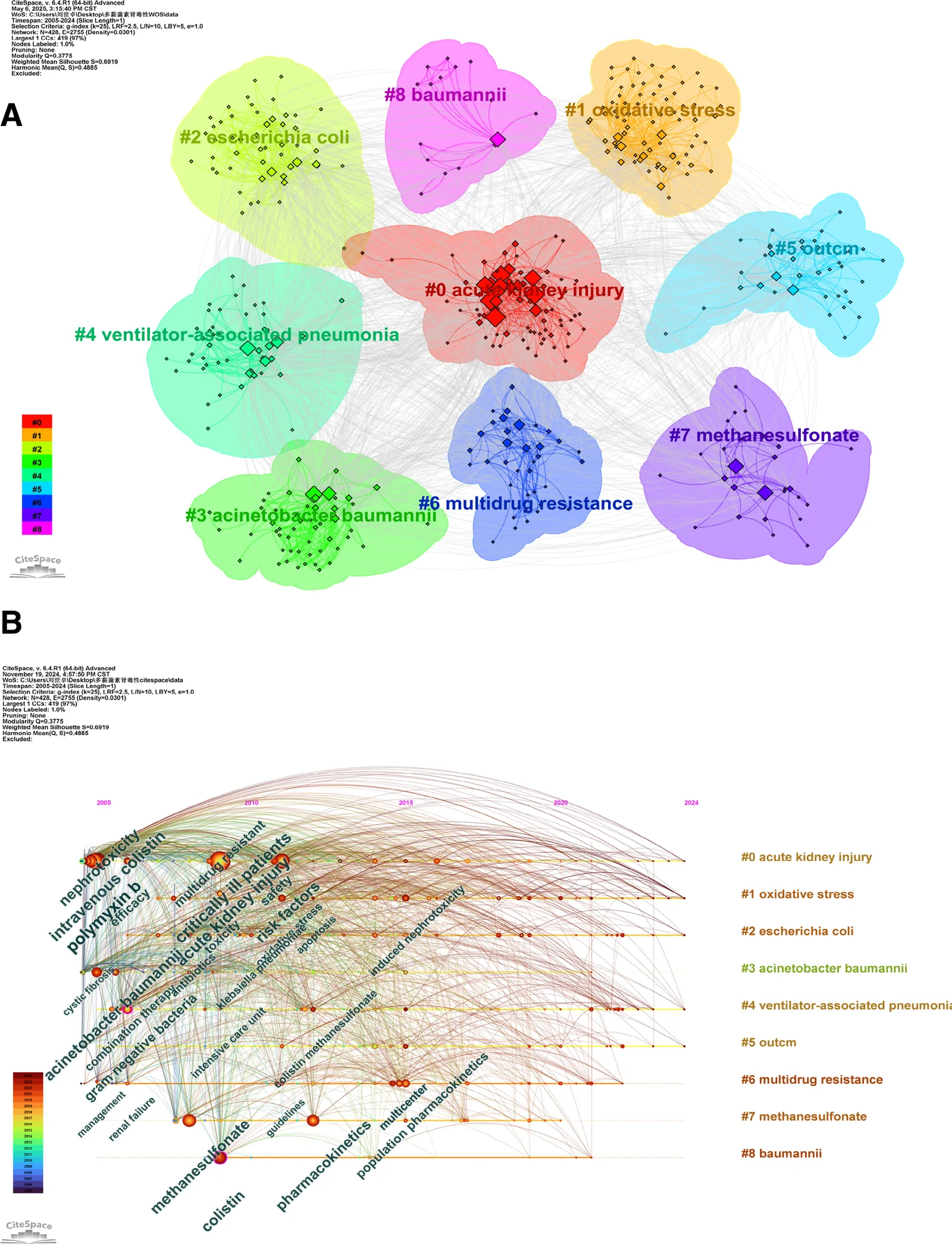
Keyword clustering analysis based on CiteSpace. (A) Keyword clustering network constructed using CiteSpace; (B) landscape view of keyword clusters generated using CiteSpace.

The clustering results showed a modularity *Q* value of 0.3775 and a silhouette score of 0.6919. The *Q* value indicated a moderately well-structured clustering network, while the silhouette score demonstrated acceptable internal consistency and homogeneity among the identified clusters. These results suggest that the keyword clustering analysis provides a reasonable representation of the major research themes and knowledge structure in the field of polymyxin-induced nephrotoxicity.

#### 3.5.3. Keyword bursts

Keyword burst analysis was performed to identify emerging research topics and temporal changes in research attention. The burst keywords identified in this study are presented in Figure 8. The duration and strength of keyword bursts reflected the changing patterns and intensity of research interest in this field.

**Figure 8.**
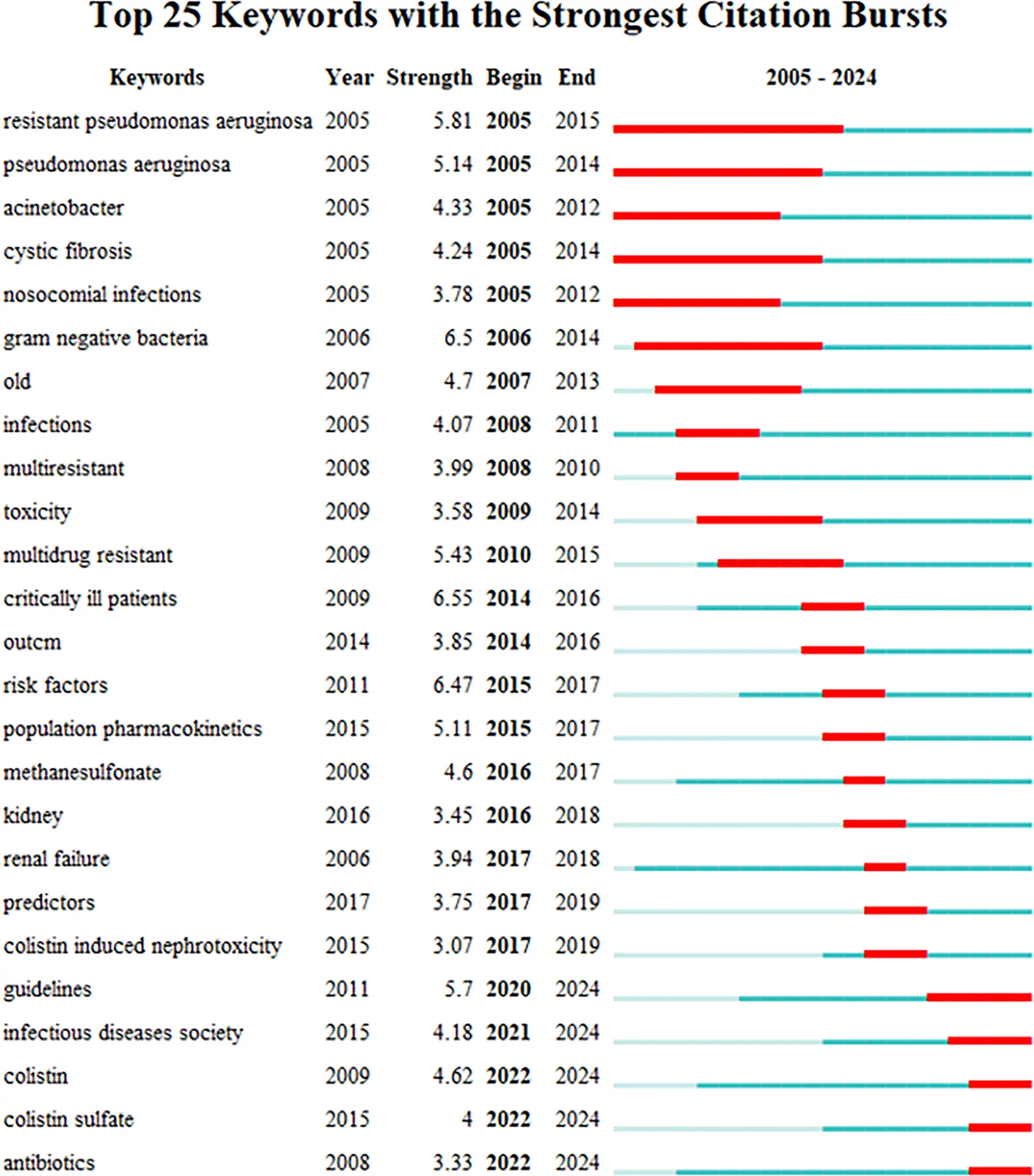
Top 25 keywords with the strongest citation bursts.

The evolution of burst keywords indicated a gradual shift in research focus over time. Early research primarily focused on infections caused by MDR Gram-negative bacteria, particularly *P aeruginosa* and *A baumannii*. Subsequently, research attention shifted toward PK/PD modeling, nephrotoxicity risk prediction, and individualized dosing strategies. In recent years, keywords related to precision medicine and treatment optimization have shown increasing prominence, suggesting emerging research directions in this field.

### 3.6. Cited literature

Table 5 presents the top 10 most cited publications related to polymyxin-induced nephrotoxicity. Among these studies, the International Consensus Guidelines for the Optimal Use of the Polymyxins, published in 2019, received the highest number of citations (n = 113). The highly cited publications mainly focused on several key topics, including the incidence and risk factors of polymyxin-associated nephrotoxicity, comparisons of nephrotoxicity between different polymyxin formulations, and PK/PD-guided dosing strategies.

**Table 5:** Top 10 most cited publications in polymyxin-induced nephrotoxicity research.

Rank	Title	Citations	Year	Journal
1	International consensus guidelines for the optimal use of the polymyxins	113	2019	PHARMACOTHERAPY
2	Population pharmacokinetics of colistin methanesulfonate and formed colistin in critically-ill patients from a multicenter study provide dosing suggestions for various categories of patients	55	2011	ANTIMICROB AGENTS CH
3	Incidence of and risk factors for colistin-associated nephrotoxicity in a large academic health system	48	2011	CLIN INFECT DIS
4	Nephrotoxicity associated with intravenous colistin (colistimethate sodium) treatment at a tertiary care medical center	43	2009	CLIN INFECT DIS
5	Risk factors for acute kidney injury in patients treated with polymyxin B or colistin methanesulfonate sodium	42	2014	INT J ANTIMICROB AG
6	In vitro assessment and multicenter cohort study of comparative nephrotoxicity rates associated with colistimethate versus polymyxin B therapy	41	2014	ANTIMICROB AGENTS CH
7	Nephrotoxicity of polymyxins: is there any difference between colistimethate and polymyxin B?	40	2017	ANTIMICROB AGENTS CH
8	High-dose, extended-interval colistin administration in critically-ill patients: is this the right dosing strategy? A preliminary study	39	2012	CLIN INFECT DIS
9	Multicenter prospective cohort study of renal failure in patients treated with colistin versus polymyxin B	37	2016	ANTIMICROB AGENTS CH
10	Higher incidence of acute kidney injury with intravenous colistimethate sodium compared with polymyxin B in critically-ill patients at a tertiary care medical center	35	2013	CLIN INFECT DIS

## 4. Discussion

This study employed bibliometric approaches to systematically characterize the developmental trends, knowledge structure, and emerging research hotspots in the field of polymyxin-induced nephrotoxicity from 2005 to 2024. The results demonstrated a generally upward trend in annual publications, reflecting increasing scientific interest in polymyxin-associated nephrotoxicity.

Keyword co-occurrence, clustering, and burst analyses revealed that research in this field has mainly concentrated on several major themes, including polymyxin safety evaluation, risk factors for acute kidney injury (AKI), underlying mechanisms of nephrotoxicity, PK/PD-guided optimization, and strategies for nephrotoxicity mitigation. Based on these bibliometric findings, the present study further explored the evolution of research themes, identified current challenges, and discussed potential future directions in this field.

It should be emphasized that bibliometric indicators, particularly citation-based metrics, primarily reflect academic attention and scientific influence rather than directly representing the methodological quality, clinical relevance, or overall impact of individual studies. Therefore, the findings of this study should be interpreted within the framework of bibliometric analysis and should not be considered a direct assessment of research quality.

### 4.1. Evolution of research themes based on bibliometric findings

#### 4.1.1. Risk factors for polymyxin-induced nephrotoxicity

Keyword co-occurrence analysis in this study identified “acute kidney injury” and “nephrotoxicity” as major research topics in the field of polymyxin-associated nephrotoxicity, highlighting the persistent interest in identifying patients at high risk of renal injury and optimizing treatment strategies. Based on the highly connected research themes revealed by the bibliometric analysis, previous studies have identified several clinical factors associated with polymyxin-induced nephrotoxicity.

Patient-related factors, including advanced age, baseline renal dysfunction, and obesity, have been reported to increase the risk of AKI.^[11–14]^ In addition, treatment-related factors, such as polymyxin dosage and concomitant administration of other nephrotoxic agents (e.g., vancomycin), have also been recognized as important contributors to nephrotoxicity risk.^[15–18]^ These findings suggest that current research has gradually shifted from simply recognizing polymyxin-associated renal toxicity toward individualized risk assessment and prevention strategies.

#### 4.1.2. Mechanistic insights into polymyxin-induced nephrotoxicity

Keyword clustering analysis in this study identified “oxidative stress” and “acute kidney injury” as major research clusters, suggesting that oxidative damage, cellular injury, and renal dysfunction have remained central themes in the investigation of polymyxin-associated nephrotoxicity. These findings indicate that mechanistic research in this field has progressively shifted from the observation of renal injury phenotypes toward elucidating the underlying cellular and molecular processes.

Previous studies have demonstrated that polymyxins can accumulate in renal tubular epithelial cells through cellular uptake processes, subsequently triggering a series of intracellular events, including oxidative stress, mitochondrial dysfunction, and apoptosis, ultimately resulting in tubular injury.^[19–24]^ Specifically, increased reactive oxygen species production, impaired antioxidant defense systems, and activation of multiple apoptotic pathways have been proposed as key mechanisms contributing to polymyxin-induced renal damage. However, the complex interactions among these injury pathways and the specific transport mechanisms regulating polymyxin accumulation in renal tissue remain incompletely understood. Further clarification of these mechanisms is essential for the development of targeted nephroprotective strategies.

#### 4.1.3. Strategies for mitigating polymyxin-associated nephrotoxicity

Keyword burst and clustering analyses in this study indicate that research on strategies to mitigate polymyxin-associated nephrotoxicity has gradually shifted from conventional dose optimization toward individualized treatment, early risk prediction, and targeted intervention approaches. Current strategies mainly focus on PK/PD-guided dosing, nephrotoxicity risk prediction models, early biomarkers, adjunctive protective approaches, and novel drug formulations.

PK/PD-based individualized dosing represents one of the earliest and most extensively investigated approaches for improving the safety profile of polymyxins. Compared with empirical dosing strategies, PK/PD-guided regimens incorporate patient-specific factors, including renal function, drug exposure, and clinical status, to optimize antimicrobial efficacy while minimizing toxicity risk.^[25]^ However, the applicability of these models remains limited by variations in patient populations and the need for further clinical validation.

With the increasing emphasis on early identification of high-risk patients, nephrotoxicity prediction models have emerged as an important research direction. Current models integrate multiple clinical variables, including polymyxin type, loading dose, infection severity, and laboratory parameters, to stratify the risk of kidney injury.^[26]^ Nevertheless, most existing models are derived from specific patient cohorts and require further validation regarding their generalizability and dynamic predictive capability.

In addition, the development of early biomarkers and nephroprotective interventions has become an emerging research focus. Conventional indicators, such as serum creatinine and blood urea nitrogen, have limited sensitivity for detecting early renal injury.^[27]^ In contrast, biomarkers including cystatin C, kidney injury molecule-1, and neutrophil gelatinase-associated lipocalin may provide earlier and more sensitive indications of renal damage.^[28]^ Meanwhile, experimental studies have suggested that antioxidant agents, anti-inflammatory compounds, and renal transporter modulators may alleviate polymyxin-induced renal injury; however, their clinical effectiveness remains to be established.

More recently, novel polymyxin formulations and targeted delivery systems have attracted increasing attention. Studies investigating hypoxia-responsive liposomal delivery systems^[29]^ and novel polymyxin analogs such as SPR206^[30]^ represent emerging strategies aimed at reducing systemic toxicity while maintaining antibacterial activity. Nevertheless, these approaches remain at an early stage, and further clinical studies are required to determine their long-term safety, efficacy, and potential for clinical translation.

### 4.2. Limitations and challenges in current research

#### 4.2.1. Gaps remain in the understanding of the mechanisms underlying polymyxin-induced nephrotoxicity

Although increasing attention has been devoted to polymyxin-induced nephrotoxicity in recent years, the underlying molecular mechanisms remain incompletely understood. In this study, keyword clustering analysis identified “oxidative stress” and “acute kidney injury” as major research themes, highlighting the continued importance of mechanistic investigations in this field.

Current evidence suggests that polymyxin-induced renal injury involves multiple interconnected processes, including oxidative stress, mitochondrial dysfunction, cellular apoptosis, and drug accumulation in renal tubular epithelial cells. However, the complex interactions among these pathways and the key determinants regulating renal accumulation of polymyxins have not yet been fully elucidated. This knowledge gap limits the development of targeted nephroprotective strategies.

Future studies should integrate advanced approaches, including multi-omics analysis, single-cell sequencing, and human-relevant experimental models, to further clarify the molecular networks involved in polymyxin-induced renal injury and identify potential therapeutic targets for preventing nephrotoxicity.

#### 4.2.2. Limitations of predictive models and dosing strategies

Keyword burst analysis in this study indicated that predictive models, PK/PD-guided dosing, and therapeutic drug monitoring (TDM) have emerged as important research directions in recent years, reflecting the growing emphasis on precision management of polymyxin therapy. However, despite these advances, current predictive models and dosing strategies still face several limitations.

Existing risk prediction models provide valuable support for identifying patients at high risk of polymyxin-induced nephrotoxicity; however, their predictive performance and clinical applicability remain limited. Most models are primarily based on baseline clinical characteristics and static laboratory parameters, which may not adequately capture dynamic changes during treatment, including fluctuations in renal function, medication adjustments, and variations in drug exposure monitored through TDM.

Similarly, although PK/PD-guided dosing strategies can optimize drug exposure and potentially reduce toxicity, their clinical implementation remains challenging due to substantial interindividual variability, changes in disease status, and limited generalizability across specific populations, such as critically-ill patients or those with complex comorbidities. Therefore, future efforts should focus on developing dynamic and individualized risk management systems that integrate real-time clinical information, drug exposure data, and novel biomarkers to improve the precision and safety of polymyxin therapy.

#### 4.2.3. Translational challenges in strategies for managing polymyxin-induced nephrotoxicity

Keyword clustering and burst analyses in this study revealed increasing research interest in nephroprotective strategies, including novel biomarkers, adjunctive interventions, and advanced drug delivery systems. However, despite the growing number of studies in these areas, significant challenges remain in translating promising experimental findings into clinically applicable approaches.

Current adjunctive therapies mainly focus on downstream injury mechanisms, such as oxidative stress, inflammation, and apoptosis, whereas interventions targeting upstream regulatory processes remain relatively limited. In addition, strategies aimed at reducing renal polymyxin accumulation through transporter modulation may be complicated by potential compensatory mechanisms involving alternative transport pathways.

For novel biomarkers, although several candidates have demonstrated potential for early detection of renal injury, their standardized measurement procedures, clinical validation, and integration into routine practice require further investigation. Similarly, while novel polymyxin formulations and targeted delivery systems have shown reduced nephrotoxicity in experimental models, their long-term safety, clinical applicability, manufacturing scalability, and cost-effectiveness require additional evaluation before widespread clinical implementation.

### 4.3. Future research directions

#### 4.3.1. Deepening mechanistic insights

Current bibliometric analyses indicate that oxidative stress and AKI remain central research clusters in this field, suggesting that mechanistic investigations continue to represent a major research focus. However, the existing literature has mainly concentrated on individual injury pathways, while the interactions among different mechanisms and the determinants of renal accumulation of polymyxins remain insufficiently characterized.

Future research should therefore further investigate the renal distribution and transport mechanisms of polymyxins by integrating multi-omics approaches, single-cell sequencing, and advanced imaging technologies. These approaches may help clarify the complex interactions among oxidative stress, inflammatory responses, mitochondrial dysfunction, and apoptotic pathways, and facilitate the identification of novel therapeutic targets for preventing polymyxin-induced nephrotoxicity.

#### 4.3.2. Advancing precision risk management

The increasing appearance of keywords related to PK/PD, TDM, and risk prediction models indicates growing interest in individualized polymyxin therapy. However, current prediction models remain limited by insufficient external validation and limited ability to incorporate dynamic treatment-related changes.

Future research should focus on developing dynamic risk management systems that integrate longitudinal clinical data, drug exposure information, renal function changes, and validated biomarkers. In addition, artificial intelligence-based approaches, including machine learning methods, may provide new opportunities for improving risk prediction and dose optimization. Nevertheless, their clinical applicability requires rigorous validation using large and representative patient populations.

#### 4.3.3. Optimizing therapeutic strategies

Bibliometric analysis indicates that research interest has gradually expanded from conventional dose optimization toward more comprehensive nephrotoxicity management strategies, including biomarker-guided monitoring, nephroprotective interventions, and targeted drug delivery approaches. However, these strategies are currently being investigated largely independently, and their incorporation into a comprehensive nephrotoxicity management framework remains limited.

Future studies should explore multidimensional strategies that integrate optimized dosing regimens, early biomarker-based monitoring, and targeted protective interventions to achieve an optimal balance between antibacterial efficacy and renal safety. In addition, the development of novel polymyxin analogs and advanced delivery systems should focus not only on reducing nephrotoxicity but also on preserving or enhancing antimicrobial activity. Well-designed clinical studies are needed to further evaluate their long-term safety, efficacy, and clinical applicability.

#### 4.3.4. Accelerating the development of safer polymyxin alternatives

The increasing attention toward novel polymyxin formulations and analogs identified through keyword burst analysis suggests that the development of safer alternatives has emerged as an important research direction.

Although early studies have demonstrated promising potential, clinical evidence supporting their long-term benefits remains limited. Future research should accelerate the development and clinical evaluation of next-generation polymyxin derivatives through well-designed preclinical and clinical studies.

These investigations should comprehensively assess their potential to reduce nephrotoxicity while maintaining antimicrobial activity, as well as evaluate resistance development, pharmacological characteristics, safety profiles, and applicability across diverse clinical settings.

### 4.4. Limitations of this study

This study has several limitations. First, the literature search was conducted exclusively using the WoSCC database, without including other widely used biomedical databases, such as Scopus and PubMed. This decision was primarily based on considerations of data consistency and methodological standardization. Different databases vary in terms of literature coverage, record formats, citation indexing systems, and keyword classification methods. The integration of multiple databases requires additional procedures, including deduplication and data harmonization, which may introduce potential heterogeneity and affect the reproducibility of bibliometric analyses. WoSCC was selected because it provides standardized bibliographic records, comprehensive citation information, and compatibility with commonly used bibliometric tools, such as CiteSpace and VOSviewer, thereby facilitating consistent data processing and analysis. Nevertheless, the use of a single database may have resulted in the omission of some relevant publications. Future studies incorporating multiple databases with rigorous standardization procedures may further enhance the comprehensiveness and robustness of bibliometric analyses.

Second, although CiteSpace and VOSviewer are widely used and validated tools for bibliometric research, the potential influence of analytical parameters, software platforms, and time slice settings on network structures and visualization results cannot be completely excluded. In this study, a 1-year time slice was adopted to capture temporal changes in research trends; however, sensitivity analyses using alternative parameters or analytical strategies were not performed. Future studies may further assess the stability of bibliometric findings through comparative analyses using different software platforms, parameter configurations, or time intervals.

Third, this study did not perform a formal methodological quality assessment of the included publications. Unlike systematic reviews and meta-analyses, bibliometric studies primarily aim to characterize research development patterns, knowledge structures, collaboration networks, and emerging trends rather than evaluate the methodological rigor of individual studies. However, variations in study quality among included publications may influence citation patterns and bibliometric indicators. Future research combining bibliometric approaches with evidence-based quality assessment methods may provide a more comprehensive understanding of the research landscape.

Finally, bibliometric indicators, including publication counts, citation frequency, and network centrality, primarily reflect academic attention and research influence rather than directly representing the methodological quality, clinical relevance, or practical impact of individual studies. In addition, citation frequency may be affected by factors such as publication age, journal visibility, and self-citation practices. Therefore, the findings of this study should be interpreted within the context of bibliometric analysis and should not be considered a direct assessment of research quality.

## 5. Conclusion

This study systematically characterized the publication trends, knowledge structure, and research hotspots of polymyxin-induced nephrotoxicity from 2005 to 2024. The results demonstrated a sustained increase in publication output over the past 2 decades, reflecting growing scientific interest in the safety challenges associated with polymyxin therapy. Keyword co-occurrence, clustering, and burst analyses revealed an evolutionary trajectory from early investigations of renal injury patterns to the identification of risk factors, elucidation of nephrotoxic mechanisms, and development of precision-based intervention strategies. Current research priorities primarily include polymyxin safety evaluation, risk factors for AKI, oxidative stress-related mechanisms, PK/PD optimization, and nephroprotective strategies.

Despite substantial advances in this field, bibliometric findings indicate several persistent challenges, including an incomplete understanding of the complex interactions underlying polymyxin-induced nephrotoxicity, limited dynamic capability and external applicability of existing risk prediction models, and insufficient clinical evidence supporting emerging nephroprotective interventions and novel formulations.

Future research should focus on addressing these knowledge gaps through the integration of advanced approaches, including multi-omics, single-cell technologies, and artificial intelligence-based analytical methods, to improve mechanistic understanding and develop more precise risk management strategies. Furthermore, continued efforts are needed to accelerate the clinical translation of safer polymyxin analogs, targeted drug delivery systems, and comprehensive nephroprotective approaches. Ultimately, the goal is to maintain the therapeutic efficacy of polymyxins while minimizing nephrotoxicity through individualized and precision medicine strategies.

## Author contributions

**Conceptualization:** Shizhuo Liu, Zexuan Tian.

**Data curation:** WenJuan DUAN, Yutian Zhang.

**Formal analysis:** WenJuan DUAN.

**Investigation:** WenJuan DUAN, Shizhuo Liu, Zexuan Tian, Linan Wang.

**Project administration:** Xiuling Yang.

**Resources:** WenJuan DUAN.

**Software:** WenJuan DUAN.

**Supervision:** Yingchao Ma.

**Visualization:** WenJuan DUAN, Shizhuo Liu.

**Writing** – **original draft:** WenJuan DUAN, Shizhuo Liu, Zexuan Tian.

**Writing** – **review & editing:** WenJuan DUAN, Linan Wang, Yutian Zhang, Yingchao Ma, Xiuling Yang.

## References

[1] Tacconelli E Carrara E Savoldi A. Discovery, research, and development of new antibiotics: the WHO priority list of antibiotic-resistant bacteria and tuberculosis. Lancet Infect Dis. 2018;18:318–27. 10.1016/S1473-3099(17)30753-3 29276051

[2] Flynn CE Guarner J. Emerging antimicrobial resistance. Mod Pathol. 2023;36:100249. 10.1016/j.modpat.2023.100249 37353202

[3] Li J Nation RL Turnidge JD. Colistin: the re-emerging antibiotic for multidrug-resistant Gram-negative bacterial infections. Lancet Infect Dis. 2006;6:589–601. 10.1016/S1473-3099(06)70580-1 16931410

[4] Zavascki AP Goldani LZ Li J Nation RL. Polymyxin B for the treatment of multidrug-resistant pathogens: a critical review. J Antimicrob Chemother. 2007;60:1206–15. 10.1093/jac/dkm357 17878146

[5] van Duin D Arias CA Komarow L. Molecular and clinical epidemiology of carbapenem-resistant Enterobacterales in the USA (CRACKLE-2): a prospective cohort study. Lancet Infect Dis. 2020;20:731–41. 10.1016/S1473-3099(19)30755-8 PMC7473597 32151332

[6] World Health Organization. Global antimicrobial resistance and use surveillance system (GLASS) report 2022. World Health Organization; 2022.

[7] Sisay M Hagos B Edessa D Tadiwos Y Mekuria AN. Polymyxin-induced nephrotoxicity and its predictors: a systematic review and meta-analysis of studies conducted using RIFLE criteria of acute kidney injury. Pharmacol Res. 2021;163:105328. 10.1016/j.phrs.2020.105328 33276108

[8] Nang SC Azad MAK Velkov T Zhou QT Li J. Rescuing the last-line polymyxins: achievements and challenges. Pharmacol Rev. 2021;73:679–728. 10.1124/pharmrev.120.000020 PMC7911091 33627412

[9] Tsuji BT Pogue JM Zavascki AP. International consensus guidelines for the optimal use of the polymyxins: endorsed by the American College of Clinical Pharmacy (ACCP), European Society of Clinical Microbiology and Infectious Diseases (ESCMID). Pharmacotherapy. 2019;39:10–39. 10.1002/phar.2209 PMC7437259 30710469

[10] Avila MP Pacheco T Arias S Bustos RH Garcia JC Jaimes D. Is there a role for the therapeutic drug monitoring of colistin? An overview. Pharmaceuticals (Basel). 2020;13:42. 10.3390/ph13030042 PMC7151705 32155714

[11] Alotaibi FM Alshehail BM Al Jamea ZAH. Incidence and risk factors of colistin-induced nephrotoxicity associated with the international consensus guidelines for the optimal use of the polymyxins: a retrospective study in a Tertiary Care Hospital, Saudi Arabia. Antibiotics (Basel). 2022;11:1569. 10.3390/antibiotics11111569 PMC9686878 36358224

[12] Sangthawan P Geater AF Naorungroj S Nikomrat P Nwabor OF Chusri S. Characteristics, influencing factors, predictive scoring system, and outcomes of the patients with nephrotoxicity associated with administration of intravenous colistin. Antibiotics (Basel). 2021;11:2. 10.3390/antibiotics11010002 PMC8772935 35052879

[13] Wu XL Long WM Lu Q. Polymyxin B-associated nephrotoxicity and its predictors: a retrospective study in carbapenem-resistant gram-negative bacterial infections. Front Pharmacol. 2022;13:672543. 10.3389/fphar.2022.672543 PMC9096016 35571125

[14] Rabi R Enaya A Sweileh MW. Comprehensive assessment of colistin induced nephrotoxicity: incidence, risk factors and time course. Infect Drug Resist. 2023;16:3007–17. 10.2147/IDR.S409964 PMC10198178 37215302

[15] Wagenlehner F Lucenteforte E Pea F. Systematic review on estimated rates of nephrotoxicity and neurotoxicity in patients treated with polymyxins. Clin Microbiol Infect. 2021;27:671–86. 10.1016/j.cmi.2020.12.009 33359542

[16] Chang K Wang H Zhao J. Risk factors for polymyxin B-associated acute kidney injury. Int J Infect Dis. 2022;117:37–44. 10.1016/j.ijid.2022.01.055 35108608

[17] Mathew SK Chapla A Venkatesan P. Genetic predisposition and high exposure to colistin in the early treatment period as independent risk factors for colistin-induced nephrotoxicity. Clin Transl Sci. 2024;17:e13764. 10.1111/cts.13764 PMC10933594 38476095

[18] Soares DS Reis ADF Junior GBDS. Polymyxin-B and vancomycin-associated acute kidney injury in critically ill patients. Pathog Glob Health. 2017;111:137–42. 10.1080/20477724.2017.1309338 PMC5445639 28353411

[19] Azad MAK Nation RL Velkov T Li J. Mechanisms of polymyxin-induced nephrotoxicity. Adv Exp Med Biol. 2019;1145:305–19. 10.1007/978-3-030-16373-0_18 31364084

[20] Xiao G Yuan F Deng Y. Polymyxin B exerts nephrotoxicity effects via inhibition of the Nrf2/NQO1 pathway-mediated antioxidant response. J Biochem Mol Toxicol. 2023;37:e23344. 10.1002/jbt.23344 36992629

[21] Dai C Li J Tang S Li J Xiao X. Colistin-induced nephrotoxicity in mice involves the mitochondrial, death receptor, and endoplasmic reticulum pathways. Antimicrob Agents Chemother. 2014;58:4075–85. 10.1128/AAC.00070-14 PMC4068542 24798292

[22] Fathalla AM Chow SH Naderer T. Polymyxin-induced cell death of human macrophage-like THP-1 and neutrophil-Like HL-60 cells associated with the activation of apoptotic pathways. Antimicrob Agents Chemother. 2020;64:e00013–20. 10.1128/AAC.00013-20 PMC7449205 32660985

[23] Azad MA Akter J Rogers KL Nation RL Velkov T Li J. Major pathways of polymyxin-induced apoptosis in rat kidney proximal tubular cells. Antimicrob Agents Chemother. 2015;59:2136–43. 10.1128/AAC.04869-14 PMC4356794 25624331

[24] Chen J Fan W Fan J. Tetrahydrocurcumin attenuates polymyxin B sulfate-induced HK-2 cells apoptosis by inhibiting endoplasmic reticulum stress-mediated PERK/eIF2α/ATF4/CHOP signaling pathway axis. Environ Toxicol. 2024;39:4995–5007. 10.1002/tox.24376 39023307

[25] Maniara BP Healy LE Doan TL. Risk of nephrotoxicity associated with nonrenally adjusted intravenous polymyxin B compared to traditional dosing. J Pharm Pract. 2020;33:287–92. 10.1177/0897190018799261 30253682

[26] Song MH Xiang BX Yang CY. A pilot clinical risk model to predict polymyxin-induced nephrotoxicity: a real-world, retrospective cohort study. J Antimicrob Chemother. 2024;79:1919–28. 10.1093/jac/dkae185 38946304

[27] Ronco C Bellomo R Kellum JA. Understanding renal functional reserve. Intensive Care Med. 2017;43:917–20. 10.1007/s00134-017-4691-6 28213622

[28] Rossiter A La A Koyner JL Forni LG. New biomarkers in acute kidney injury. Crit Rev Clin Lab Sci. 2024;61:23–44. 10.1080/10408363.2023.2242481 37668397

[29] Liu F Zou L Chen Y. A polymyxin B loaded hypoxia-responsive liposome with improved biosafety for efficient eradication of bacterial biofilms. Nano Res. 2024;17:8325–36.

[30] Bruss JB Bader J Hamed KA. Safety and pharmacokinetics of SPR206 in subjects with varying degrees of renal impairment. Antimicrob Agents Chemother. 2023;67:e0050523–23. 10.1128/aac.00505-23 PMC10648942 37823647

